# Comparison of Anesthesia Times and Billing Patterns by Anesthesia Practitioners

**DOI:** 10.1001/jamanetworkopen.2018.4288

**Published:** 2018-11-09

**Authors:** Eric C. Sun, Richard P. Dutton, Anupam B. Jena

**Affiliations:** 1 Department of Anesthesiology, Perioperative and Pain Medicine, Stanford University School of Medicine, Stanford University, Stanford, California; 2 US Anesthesia Partners, Department of Anesthesiology, Texas A&M School of Medicine, Bryan; 3 Department of Health Care Policy, Harvard Medical School, Boston, Massachusetts; 4 Department of Medicine, Massachusetts General Hospital, Boston; 5 National Bureau of Economic Research, Cambridge, Massachusetts

## Abstract

**Importance:**

Most physicians must exercise discretion in choosing billing details that determine payment for their services. Understanding the degree to which physicians inappropriately use this discretion has important implications for payment policies. However, separating higher case complexity from inappropriate billing has made this a challenging issue to study. Anesthesia offers a useful test case because practitioners are partly compensated by self-reported length of time (anesthesia time) spent on a case.

**Objective:**

To characterize the incidence and consequences of inappropriate billing practices among anesthesia practitioners.

**Design, Setting, and Participants:**

In this cross-sectional study of data from a large anesthesia registry, 6 261 955 procedures performed by 4221 anesthesia practitioners (physician anesthesiologists, nurse anesthetists, and anesthesiologist assistants) between January 1, 2010, and March 31, 2015, were studied. A total of 3047 practitioners practiced primarily in community hospitals, whereas 453 practiced primarily in university hospitals and 721 practiced in other settings (eg, specialty hospital).

**Exposures:**

Practitioners with anomalous patterns were identified as those reporting an unusually high number of anesthesia times ending in a multiple of 5 minutes (eg, 65 minutes).

**Main Outcomes and Measures:**

Incidence of anomalous patterns among anesthesia practitioners and the increase in anesthesia times associated with these patterns.

**Results:**

This study included 4221 practitioners who each performed at least 300 anesthetic procedures. Practitioners in the top fifth percentile reported anesthesia times ending in a multiple of 5 minutes a mean (SD) of 53.7% (13.7%) of the time (range, 36.8%-96.1%), whereas practitioners in the 6th to 10th percentiles reported anesthesia times ending in a multiple of 5 minutes a mean (SD) of 31.8% (2.0%) of the time (range, 29.2%-36.7%). Practitioners in the top fifth percentile submitted billing for anesthesia times that exceeded the expected time by a mean of 21.5 minutes (95% CI, 15.8-27.1 minutes).

**Conclusions and Relevance:**

In this study, findings suggest that anesthesia practitioners with the highest tendency to report anesthesia times ending in a multiple of 5 minutes did so with high frequency, which reflects anomalous billing. These practitioners also sought payment for longer-than-expected anesthesia times, which would correspond to higher payment for their services.

## Key Points

**Question:**

What is the incidence of anomalous billing among anesthesia practitioners in the United States?

**Findings:**

In this cross-sectional study of 4221 anesthesia practitioners in the United States, 212 reported an unusually large number of cases with durations that were a perfect multiple of 5 minutes. These practitioners submitted billing for anesthesia times that exceeded the expected time by a mean of 21.5 minutes.

**Meaning:**

Anesthesia practitioners with the highest tendency to report anesthesia times with a demonstrable anomaly (times rounded to 5 minutes) may be more likely to report longer-than-expected anesthesia times.

## Teaser

This cross-sectional study analyzes data from the National Anesthesia Clinical Outcomes Registry to determine the incidence of anomalous billing among anesthesia practitioners in the United States.

## Introduction

In the United States, hospitals and health care practitioners exercise discretion in determining the amounts paid for their services. For example, in outpatient settings, payment is often based on the practitioner’s assessment of the complexity of the patient’s case and the issues addressed. Although insurers provide this discretion because complex cases require more time, there are concerns that it may be used inappropriately to increase compensation. For example, in response to the elimination of consultation payments from the Medicare Part B Physician Fee Schedule in 2010, one study^1^ demonstrated that practitioners nearly fully substituted toward billing for more expensive new office visits, suggesting inappropriate use of subjective codes in the fee schedule. More generally, studies suggest that some physicians engage in revenue-maximizing behavior, as exemplified by supplier-induced demand^2,3,4^ and self-referral.^5,6^ Characterizing the degree to which practitioners inappropriately use their discretion has important policy implications. If inappropriate discretion is widespread, this would argue in favor of payment mechanisms with reduced discretion. In addition, at its extreme, inappropriate use of discretion constitutes insurance fraud, which imposes significant costs. In 2014, $1.4 billion was spent to combat Medicare and Medicaid fraud,^7^ and the cost of fraud more generally has been estimated to range from $82 billion to $272 billion.^8^

Previous studies have demonstrated the presence of inappropriate billing practices among hospitals^9^ and insurers^10^ and have found that hospitals modify behavior in response to antifraud enforcement efforts.^11^ However, fewer studies have examined practitioner behavior. A prior study^12^ found regional variation in the frequency of diagnosis codes among Medicare beneficiaries but did not address the implications for billing. Moreover, this study demonstrated a key difficulty in assessing the degree of inappropriate discretion: if one practitioner states that a particular case is complex and another does not, who is correct? Although researchers and insurers can identify practitioners who bill anomalously, such as billing an unusually high number of complex cases, it is difficult to identify whether the anomaly is attributable to inappropriate discretion or a truly higher incidence of complex cases, particularly because there are often few objective criteria to judge complexity.

In the United States, the amount that insurers pay for a given anesthetic case is based on the number of anesthesia units it generates. Each case is associated with a fixed number of units based on type of surgery; for example, in 2014, a laparoscopic cholecystectomy generated 7 units for fee-for-service Medicare patients.^13^ In addition, a case generates units based on the self-reported amount of time spent providing care (anesthesia time), earning 1 unit for every 15 minutes.^14^ Insurer regulations typically dictate that anesthesia time starts when the anesthesia practitioner begins preparing the patient for the procedure and ends when the patient is transferred to postanesthesia care. Because many insurers pay to the actual minute (eg, a 12-minute case earns 0.8 unit), insurers require that practitioners report exact times without any rounding. In a 2014 survey conducted by the American Society of Anesthesiologists, the median payment for an anesthesia unit among commercial payers was $66, whereas the national Medicare rate was $22.62.^15^

In the United States, anesthesia care can be provided by anesthesiologists (physicians trained in the specialty of anesthesiology), nurse anesthetists, or anesthesiologist assistants, with the last 2 groups typically providing care under the supervision of an anesthesiologist.^16^ Throughout this article, the term *anesthesia practitioner* is used to refer to all 3 groups.

In this study, we examined the incidence and consequences of inappropriate discretion in billing in the case of anesthesia. Anesthesia presents a unique case because practitioners are paid in large part by the self-reported amount of time that they spend on a given case (anesthesia time), giving an incentive to report longer anesthesia times. Similar to other specialties, identifying practitioners with anomalously long anesthesia times is not sufficient to demonstrate inappropriate discretion because these anomalous times could be explained by unobserved clinical or institutional factors. However, anesthesia is unique because other anomalies, such as an excess number of cases with an anesthesia time ending in a multiple of 5 minutes (eg, reporting an excess number of cases with an anesthesia time of 75 minutes as opposed to 74 or 76 minutes), have no plausible clinical basis. Thus, it is possible to identify inappropriate discretion through a 2-step process. First, use the presence of anomalous billing patterns with no clinical basis, such as an excess number of cases with an anesthesia time ending in a multiple of 5 minutes, to identify practitioners who may be billing anomalously. Second, because rounding habits may explain this former phenomenon, estimate whether these practitioners report longer-than-expected anesthesia times based on observable clinical and institutional characteristics. To the extent that practitioners with anomalous patterns also report anesthesia times that are longer than would be expected, this would argue that they may be inappropriately using their discretion. This approach, which uses statistical anomalies to identify anomalous behaviors, has been used in other settings to identify anomalous behaviors (eg, cheating on standardized examinations^17^). We applied this approach using a nationwide US registry of anesthesia cases to characterize the scope of anomalous and inappropriate billing practices among anesthesia practitioners.

## Methods

We used deidentified data from the National Anesthesia Clinical Outcomes Registry (NACOR), a registry of anesthesia cases that is maintained by the Anesthesia Quality Institute.^18^ The registry is a collection of anesthesia claims that are provided by participating anesthesia practices (283 practices as of April 2015). The database includes information obtained from billing and medical records that are converted into a publicly available file, the Participant User File. For each case, the Participant User File provides information, such as surgical and anesthesia *Current Procedural Terminology* (*CPT*) codes, diagnosis codes (*International Classification of Diseases, Ninth Revision* [*ICD-9*]), and the reported anesthesia time. The reported anesthesia time in NACOR is extracted from administrative records and represents the same time that was sent to the insurer to establish payment. In addition, the data report encrypted identifiers for the specific facility, anesthesia group, and anesthesia practitioner. Because anesthesia practices report these data to varying extents, not all data are available for every case. NACOR data have been extensively used for outcomes research in anesthesiology.^19,20^ This study followed the Strengthening the Reporting of Observational Studies in Epidemiology (STROBE) reporting guideline for reporting cross-sectional study results. Institutional review board review was not required according to the Stanford University protocol for deidentified data.

The data included 26 568 734 anesthesia cases that occurred between January 1, 2010, and March 31, 2015. We excluded cases for which the following variables were missing: anesthesia time (n = 1 888 625), surgical *CPT* code (n = 6 359 104), patient age (n = 269 761), patient sex (n = 441 368), specific anesthesia practitioner (n = 880 132), and *ICD-9* diagnosis codes (n = 425 750). NACOR classifies facilities into 9 categories (university hospital, large community hospital, medium community hospital, small community hospital, specialty hospital, attached surgery center, freestanding surgery center, pain clinic, and surgeon’s office); we excluded cases for which the facility was unknown or listed as a pain clinic or surgeon’s office (n = 3 819 101). We excluded cases with more than 1 practitioner (which typically occurs when 1 practitioner relieves another later in the day, n = 5 047 903). Finally, we restricted analyses to surgical *CPT* codes with at least 1000 observations and practitioners who had performed at least 300 procedures, resulting in a final sample of 6 261 955 anesthetic cases. These procedures were performed at 931 surgical facilities and encompass 819 surgical *CPT* codes. Anesthesia care in these cases was provided by 4221 anesthesia practitioners who were employed by 147 anesthesia practices. The number of facilities is larger than the number of anesthesia practices because many practices will cover more than 1 facility (eg, a practice may cover a hospital and several surgery centers).

### Statistical Analysis

Identifying practitioners with anomalously long anesthesia times is not sufficient to measure inappropriate discretion because these times may be explained by unobserved clinical or institutional factors and differences in rounding habits. Therefore, we used a 2-step process to estimate the incidence and consequences of inappropriate billing discretion. First, we identified practitioners with anomalous patterns of anesthesia times (those reporting an excess number of anesthesia times ending in a multiple of 5 minutes) for which there can be no clinical justification. Second, we identified whether these anomalous practitioners also tended to report longer anesthesia times than their peers nationally after adjusting for type of surgery, surgical facility, and patient characteristics. As a first step, for each practitioner, we calculated the proportion of anesthesia times ending in a multiple of 5 minutes. We then ranked practitioners based on the percentage of cases ending in a multiple of 5 minutes and identified practitioners in the top 5th percentile and the top 6th to 10th percentiles. Simple summary statistics regarding patient and practitioner characteristics were calculated for each of these 3 groups (top 5th percentile, top 6th to 10th percentiles, and remaining practitioners) by using a 2-tailed *t* test to assess for statistical significance in the case of continuous variables and a χ^2^ test for discrete (yes/no) variables.

Rounding anesthesia times to the nearest 5 minutes may be anomalous but is not necessarily indicative of inappropriately high billing (eg, practitioners could be rounding down). To assess this possibility, we analyzed whether practitioners with an unusually high proportion of anesthesia times rounded to the nearest 5 minutes also had anesthesia times that were longer than their peers after adjustment for surgery type, surgical facility, and patient characteristics. Specifically, we used multivariable linear regression to estimate expected anesthesia times for each case. Independent variables included indicators for type of surgery (based on surgical *CPT* code), indicators for facility, patient age and sex, and indicators for patient comorbidities based on *ICD-9* codes (full list of comorbidities and *ICD-9* codes is given in eTable 1 in the Supplement). By incorporating facility-specific indicators, we essentially compared a given practitioner’s times against the times of other practitioners at the same facility. An advantage of this approach is that it is robust to facility-specific factors (such as speed of the operating room staff and the surgical team) that may be associated with anesthesia times.

We then calculated the difference between the observed time and the expected time for each case. Linear regression was used to estimate the extent to which those practitioners who were most likely to report anesthesia times ending in a multiple of 5 minutes (ie, practitioners in the top 5th percentile and the top 6th to 10th percentile) were also more likely to exceed their expected times. All analyses were performed using Stata statistical software, version 14.0 (StataCorp). Details of our regression model can be found in the eMethods and eTable 2 in the Supplement. Because our primary analysis examined differences in outcomes between 2 groups (the top 5th percentile and the top 6th to 10th percentile of practitioners) against the remaining practitioners, we defined 2-sided *P* ≤ .025 as indicating statistical significance.

### Additional Analyses

We conducted additional analyses to determine the robustness of our results across several subgroups. We examined 3 subgroups based on the type of facility (university hospital, community hospital, and specialty hospital or surgery center) and subgroups based on whether an anesthesia resident was involved in the case. For each subgroup analysis, we reranked practitioners within the subgroup based on frequency of cases with anesthesia times ending in 5 minutes (ie, top fifth percentile) and performed the analyses described above again.

Given the difference between the initial data set (n = 26 568 734) and final sample (n = 6 261 955), we performed several comparisons between the final sample and the cases that were dropped to characterize the extent to which the final sample is representative of the overall data set. For the cases for which we had data on facility type (n = 20 217 215), we compared the distribution of facility type (eg, university hospital) between the final sample and the excluded cases. We also compared the distribution of surgical *CPT* codes (ie, surgery type) between the included and excluded cases for the cases with nonmissing surgical *CPT* codes (n = 19 554 872).

## Results

**Figure 1.**
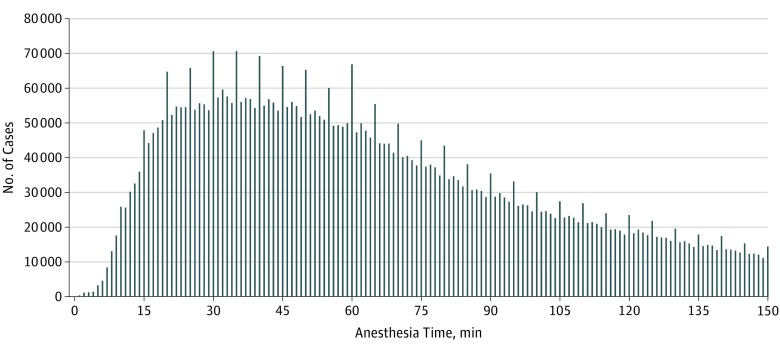
Distribution of Anesthesia Times for the 6 261 955 Cases in the Study Sample The figure is truncated at 150 minutes; for times past 150 minutes, see eFigure in the Supplement.

This study included 4221 practitioners who each performed at least 300 anesthetic procedures. The mean (SD) anesthesia time was 106 (129) minutes, and the median (range) was 68 minutes (1-1439 minutes). Anesthesia times that were a multiple of 5 minutes were unusually prevalent, as shown by increases in the distribution that occur every 5 minutes (Figure 1). For example, 66 920 cases had an anesthesia time of 60 minutes compared with 49 985 with an anesthesia time of 59 minutes and 47 331 with an anesthesia time of 61 minutes. The general pattern remained the same when all procedures were considered (eFigure in the Supplement). Similarly, 802 practitioners (19.0%) reported a percentage of anesthesia times ending in 0 or 5 that were statistically distinguishable from the expected value of 20%, using a threshold of *P* < .001 for statistical significance. Anesthesia times that were a multiple of 5 minutes were longer vs all other cases (mean, 111 minutes [95% CI, 110-112 minutes] vs 104 minutes [95% CI, 104-104 minutes]). A total of 3047 practitioners (72.2%) practiced in a community hospital, whereas 453 (10.7%) practiced in a university hospital, with the remaining practitioners (721 [17.1%]) practicing in other settings (eg, specialty hospitals). Table 1 presents additional characteristics of our sample, stratified by 3 groups of practitioners (top 5th percentile in terms of reporting anesthesia times ending in a multiple of 5 minutes, top 6th to 10th percentile, and remaining practitioners). Compared with the remaining practitioners, those in the top fifth percentile were more likely to practice in university hospitals (50 [23.6%] vs 347 [9.1%]; *P* < .001), less likely to practice in specialty hospitals or surgical centers (12 [5.7%] vs 694 [18.2%]; *P* < .001), and more likely to practice in the northeastern United States (111 [52.4%] vs 377 [9.9%]; *P* < .001). On average, practitioners in the top fifth percentile were less likely to treat male patients (38.7%; *P* < .001) and patients with depression (1.1%; *P* = .03) and were more likely to encounter patients with congestive heart failure (1.0%), hypertension (11.9%), diabetes (5.1%), and chronic kidney disease (2.4%) (*P* < .001 for all these comorbidities). These comparisons were similar for practitioners in the 6th to 10th percentile. For example, practitioners in the 6th to 10th percentile were also more likely to practice in university hospitals (56 [26.5%] vs 347 [9.1%]). 

**Table 1.** Sample Summary Statistics According to Percentiles Based on the Cases With Anesthesia Times Ending in a Multiple of 5

Variable	Top Fifth Percentile (n = 212)		Top 6th to 10th Percentiles (n = 211)		Remaining Practitioners (n = 3798)
	Practitioners	*P* Value^a^	Practitioners	*P* Value^a^	
Type of institution, No. (%) [SE]					
University hospital	50 (23.6) [2.9]	<.001	56 (26.5) [3.0]	<.001	347 (9.1) [0.5]
Community hospital	150 (70.7) [3.1]	.56	140 (66.4) [3.3]	.05	2757 (72.6) [0.7]
Specialty hospitals and surgery centers	12 (5.7) [1.6]	<.001	15 (7.1) [1.8]	<.001	694 (18.2) [0.6]
US Census region, No. (%) [SE]					
Northeast	111 (52.4) [3.4]	<.001	39 (18.5) [2.7]	<.001	377 (9.9) [0.5]
Midwest	35 (16.5) [2.6]	<.001	93 (44.1) [3.4]	<.001	1119 (29.4) [0.7]
South	36 (17.0) [2.6]	<.001	45 (21.3) [2.8]	.02	1101 (29.0) [0.7]
West	30 (14.2) [2.4]	<.001	34 (16.1) [2.5]	<.001	1201 (31.6) [0.8]
Patient characteristics, mean [SE], %					
Age, y	50.8 [0.6]	.47	52.1 [0.6]	.02	50.2 [0.2]
Male	38.7 [0.7]	<.001	41.9 [0.7]	.31	42.5 [0.1]
Patient comorbidities, mean [SE], %					
Congestive heart failure	1.0 [0.1]	<.001	0.8 [0.1]	.09	0.7 [0.02]
Peripheral vascular disease	0.8 [0.1]	.21	1.0 [0.2]	.02	0.7 [0.02]
Hypertension	11.9 [0.9]	<.001	5.7 [0.6]	.27	5.1 [0.1]
Chronic obstructive pulmonary disease	0	.41	0	.63	0
Diabetes	5.1 [0.4]	<.001	3.0 [0.3]	.006	2.3 [0.1]
Chronic kidney disease	2.4 [0.2]	<.001	1.6 [0.1]	<.001	1.2 [0.0]
Cancer	3.9 [0.2]	.59	4.8 [0.3]	.002	4.1 [0.1]
Cerebrovascular disease	0.4 [0.04]	.06	0.4 [0.04]	.11	0.3 [0.01]
Dementia	0.01 [0.0]	.26	0.002 [0.001]	.05	0.01 [0.001]
Myocardial infarction	0.2 [0.04]	.76	0.2 [0.03]	.98	0.2 [0.01]
Liver disease	0.4 [0.06]	.74	0.6 [0.07]	.007	0.4 [0.01]
Alcohol abuse	0.1 [0.01]	.62	0.08 [0.01]	.69	0.08 [0.003]
Drug abuse	0.05 [0.01]	.44	0.03 [0.01]	.31	0.04 [0.002]
Schizophrenia	0.07 [0.01]	.13	0.1 [0.06]	.43	0.2 [0.02]
Depression	1.1 [0.2]	.03	1.3 [0.3]	.13	1.9 [0.09]

^a^ *P* values represent the statistical significance of the difference between the given group and remaining practitioners.

**Figure 2.**
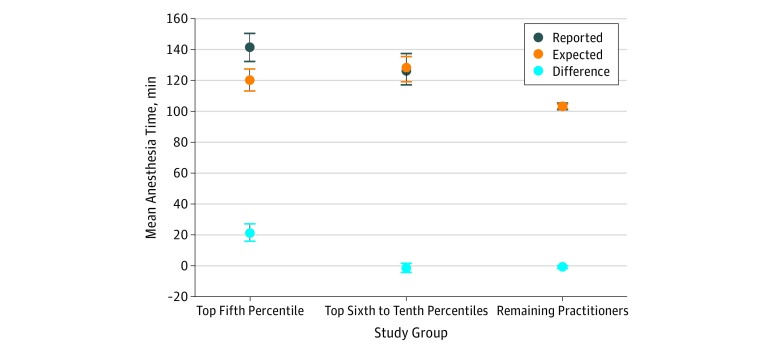
Reported and Expected Anesthesia Times and the Mean Difference Between the These Times for Each Study Group Anesthesia practitioners were classified into 1 of 3 groups based on the extent to which their reported anesthesia times ended in a multiple of 5 minutes (top fifth percentile, top sixth to tenth percentiles, and remaining practitioners). Error bars with 95% CIs are shown and are corrected for clustering within practitioners.

Practitioners in the top 5th percentile reported anesthesia times ending in a multiple of 5 minutes a mean (SD) of 53.7% (13.7%) of the time (range, 36.8%-96.1%), whereas practitioners in the 6th to 10th percentile reported anesthesia times ending in a multiple of 5 minutes a mean (SD) of 31.8% (2.0%) of the time (range, 29.2%-36.7%). After adjustment for the hospital where the procedure occurred, surgery type, patient comorbidities, age, and sex, practitioners in the top 5th percentile reported anesthesia times that exceeded the expected time by a mean of 21.5 minutes (95% CI, 15.8-27.1 minutes; mean, 141 minutes [95% CI, 132-151 minutes] vs 120 minutes [95% CI, 112-128 minutes]) (Figure 2). By contrast, practitioners in the top 6th to 10th percentile had observed anesthesia times that were similar in length to the expected time (mean, 126 minutes [95% CI, 117-136 minutes] vs 128 minutes [95% CI, 118-137 minutes]; mean difference, −1.3 minutes; 95% CI, −4.3 to 1.6 minutes; *P* = .38). The remaining practitioners reported anesthesia times that were similar to the predicted time (mean, 102 minutes [95% CI, 101-104 minutes] vs 103 minutes [95% CI, 102-105 minutes]; mean difference, −0.8 minute; 95% CI, –1.5 to −0.1; *P* = .02).

These results largely persisted across each of the subgroups that we examined (Table 2). For example, for practitioners in university hospitals, the top fifth percentile reported anesthesia times that exceeded the expected time by a mean of 26.5 minutes (95% CI, 19.6-33.2 minutes; *P* < .001), whereas the top fifth percentile of practitioners in community hospitals exceeded expected times by a mean of 16.5 minutes (95% CI, 9.2-23.9 minutes; *P* < .001). Finally, we also examined differences in some observable characteristics (eg, type of facility and distribution of surgical *CPT* codes) between the cases in our final sample and excluded cases. Although our results suggested that anesthesia practitioners in the final sample were more likely to practice in medium-sized community hospitals (2 706 688 [43.2%] vs 5 633 804 [40.4%]) and specialty hospitals (234 487 [3.7%] vs 278 303 [2.0%]) and less likely to practice in other types of facilities, these differences were small (eTable 3 in the Supplement). Similarly, the types of procedures for both groups were qualitatively similar (eTable 4 in the Supplement).

**Table 2.** Difference Between Observed and Expected Anesthesia Times (Subgroup Analyses)

Group	Top Fifth Percentile (n = 212)		Top 6th to 10th Percentiles (n = 211)		Remaining Practitioners (n = 3798)
	Observed Minus Expected Anesthesia Time, Mean (95% CI), min	*P* Value^a^	Observed Minus Expected Anesthesia Time, Mean (95% CI), min	*P* Value^a^	Observed Minus Expected Anesthesia Time, Mean (95% CI), min
Facility type					
University	26.5 (19.6 to 33.2)	<.001	−4.9 (−10.2 to 0.4)	.75	−5.9 (−8.9 to −2.9)
Community	16.5 (9.2 to 23.9)	<.001	0.2 (−3.7 to 4.1)	.91	0.4 (−0.4 to 1.3)
Other (specialty hospital or surgical center)	38.8 (20.6 to 56.9)	<.001	−0.9 (−3.6 to 1.7)	.36	−2.2 (−2.9 to −1.5)
Presence of anesthesia resident					
Yes	31.4 (24.2 to 38.6)	<.001	−3.1 (−7.9 to 1.7)	.21	−4.0 (−6.4 to −1.6)
No	22.5 (10.0 to 35.0)	<.001	−1.2 (−7.4 to 4.9)	.90	−0.8 (−1.8 to 0.2)

^a^ *P* values represent the statistical significance of the difference between the given group and remaining practitioners.

## Discussion

Physicians are often paid for services for which complexity is tied to compensation and that rely on physician discretion in reporting. Identifying the extent to which physicians inappropriately use their discretion is important in designing optimal payment policy but is difficult to study because complexity is often measurable only by the physician. In this study, we found that some anesthesia practitioners seemed to inappropriately exercise their discretion in billing, as suggested by reporting anesthesia times that were disproportionately a multiple of 5 minutes. Rounding to the nearest 5 minutes alone would not significantly affect the total case time, but it could suggest a proclivity for other forms of inaccurate reporting. We found that practitioners with a propensity to round their times also reported anesthesia times 22 minutes longer than expected, corresponding to increased revenue ranging from $34 to $98 per case based on reimbursements by various payers.^15^ This 22-minute increase represents a 21% increase in time-related payment associated with the mean case and a 32% increase associated with the median case in our sample. Subgroup analyses revealed that anomalous billing patterns were associated with increased case length across a variety of practice settings (eg, community and university hospitals), and the association was particularly strong at specialty hospitals and surgery centers, a finding that is arguably consistent with concerns about increased costs for operations performed in specialty surgical hospitals.^21^

Our findings are not necessarily conclusive of inappropriate billing. It is possible that those practitioners with a disproportionate share of anomalous billing report anesthesia times that are closer to reality, whereas other practitioners may systematically report anesthesia times that are less than the actual time that could justifiably be billed. The likelihood of this possibility seems low, however, given that it would suggest that most practitioners tend to underreport anesthesia times rather than the alternative possibility that a few practitioners inappropriately overstate anesthesia times.

Our results have important policy implications. Like other studies,^9,10,11,12^ our study suggests potential cost savings from reducing the amount of discretion that health care practitioners have in determining the payment that they receive for a given service. In anesthesia specifically, our results suggest that paying practitioners based solely on the type of case performed (and removing the time element) may be a better alternative to current payment policy. Under this new policy, anesthesiologists would no longer be paid based on the self-reported amount of time spent on the case, but similar to surgeons, the policy could allow anesthesiologists to add a modifier code for particularly difficult cases. Another potential policy would be to explicitly tie the start and stop of anesthesia time to surgical times, such as the times when the patient enters and exits the operating room, which are typically recorded by a third party (the operating room staff).

### Limitations

Our results should be viewed in light of their limitations. Although we adjusted for surgery type, surgical facility, and patient characteristics, we cannot rule out the possibility that other unobserved factors could explain why practitioners with anomalous anesthesia times report longer times. In particular, our data set did not have patient-specific identifiers; thus, we could not use patient fixed effects to adjust for unobservable patient characteristics. However, it is unclear how unobserved patient characteristics would be correlated with billing anomalies. Moreover, our subgroup analyses revealed no substantial differences among procedures performed by an anesthesia resident, a proxy for case complexity, compared with those performed by a practitioner other than an anesthesia resident.

Our data did not include information on specific practitioners; thus, we were unable to discern the degree to which anomalies were more common among anesthesiologists than among nurse anesthetists. We also only analyzed a fairly obvious billing anomaly. Our approach would miss many other forms of inappropriate billing, such as adding a fixed number of minutes to each case. Moreover, our approach compared a given practitioner’s times against the anesthesia times reported by other practitioners practicing in the same facility. Although this approach is robust to facility-specific factors (such as speed of the operating room teams), it tends to underestimate the degree of inappropriate discretion to the extent that all practitioners at a given facility use inappropriate discretion or to the extent that inappropriate discretion occurs at the system level (eg, at the billing office). Our results were based on a subset of cases from practices reporting data to NACOR and may not generalize to other populations. However, although there were a large number of excluded cases, our sensitivity analyses suggest that the excluded cases were qualitatively similar in many ways to the included cases. In addition, data submitted to NACOR are estimated to account for 25% of all anesthesia cases in the United States.^22^ Finally, our findings should not be used to indicate fraud because we are unable to ascertain intent.

## Conclusions

Our results suggest that concerns over whether health care practitioners may inappropriately use their discretion to set payments are not unwarranted because we speculate that anesthesia practitioners are not unique in the scale or scope of their behaviors. Given our findings, we suggest that future studies should examine the degree to which physicians and other health care practitioners inappropriately use their discretion in determining reimbursement, as well as the potential effect of alternative payment policies.

## Notes

Supplement. **eTable 1.** List of Medical Comorbidities and ICD-9 Codes **eTable 2.** Regression Coefficients for Case Prediction **eTable 3.** Differences in Type of Institution Between Included and Excluded Cases **eTable 4.** Distribution of Surgical CPTs Between Included and Excluded Cases **eMethods.** Regression Model **eFigure.** Distribution of Anesthesia Times
