# Supplementary material for: Comparison of Anesthesia Times and Billing Patterns by Anesthesia Practitioners
Source: JAMA Netw Open. 2018 Nov 9;1(7):e184288. doi: 10.1001/jamanetworkopen.2018.4288 (PMC6324364; doi:10.1001/jamanetworkopen.2018.4288)
Supplement: Supplement. — eTable 1. List of Medical Comorbidities and ICD-9 Codes eTable 2. Regression Coefficients for Case Prediction eTable 3. Differences in Type of Institution Between Included and Excluded Cases eTable 4. Distribution of Surgical CPTs Between Included and Excluded Cases eMethods. Regression Model eFigure. Distribution of Anesthesia Times [file jamanetwopen-1-e184288-s001.pdf]

Supplementary Online Content

Sun EC, Dutton RP, Jena AB. Comparison of anesthesia times and billing patterns by anesthesia practitioners. *JAMA Netw Open.* 2018;1(7):e184288. doi:10.1001/jamanetworkopen.2018.4288

eTable 1. List of Medical Comorbidities and ICD-9 Codes

eTable 2. Regression Coefficients for Case Prediction

eTable 3. Differences in Type of Institution Between Included and Excluded Cases

eTable 4. Distribution of Surgical CPTs Between Included and Excluded Cases

eMethods. Regression Model

eFigure. Distribution of Anesthesia Times

This supplementary material has been provided by the authors to give readers additional information about their work.

© 2018 Sun EC et al. *JAMA Netw Open.*

eTable 1. List of Medical Comorbidities and ICD-9 Codes

Congestive Heart Failure	398.91, 402.01, 402.11, 402.91, 404.01, 404.03, 404.11, 404.13, 404.91, 404.93, 425.4-425.9, 428.x
Peripheral Vascular Disease	093.0, 437.3, 440.x, 441.x, 443.1-443.9, 447.1, 557.1, 557.9, V43.4
Hypertension	401-405
Chronic Obstructive Pulmonary Disease	416.8, 416.9, 490.x-505.x, 506.4, 508.1, 508.8
Diabetes Mellitus	250.x
Chronic Kidney Disease	403.01, 403.11, 403.91, 404.02, 404.03, 404.12, 404.13, 404.92, 404.93, 585.x, 586.x, 588.0, V42.0, V45.1, V56.x
Cancer	140.x-172.x, 174.x-202.x, 203.0, 238.6
Cereberovascular Disease	430.x-438.x
Myocardial Infarction	410.x, 412.x
Dementia	290.x
Liver Disease	070.22, 070.23, 070.32, 070.33, 070.44, 070.54, 070.6, 070.9, 456.0-456.2, 570.x, 571.x, 572.2-572.8, 573.3, 573.4, 573.8, 573.9, V42.7
Alcohol Abuse	265.2, 291.1-291.3, 291.5-291.9, 303.0, 303.9, 305.0, 357.5, 425.5, 535.3, 571.0-571.3, 980.x, V11.3
Drug Abuse	292.x, 304.x, 305.2-305.9, V65.42
Schizophrenia	293.8, 295.x, 296.04, 296.14, 296.44, 296.54, 297.x, 298.x
Depression	296.2, 296.3, 296.5, 300.4, 309.x, 311

© 2018 Sun EC et al. *JAMA Netw Open.*

eTable 2. Regression Coefficients for Case Prediction[c]

	Regression Coefficient	p
Patient Comorbidities		
Congestive Heart Failure	0.024 (0.013 to 0.033)	<0.001
Peripheral Vascular Disease	0.036 (0.022 to 0.049)	<0.001
Hypertension	0.012 (0.006 to 0.017)	<0.001
Chronic Obstructive Pulmonary Disease	0.12 (-0.07 to 0.31)	0.22
Diabetes Mellitus	0.021 (0.016-0.026)	<0.001
Chronic Kidney Disease	0.022 (0.010-0.034)	<0.001
Cancer	0.11 (0.11-0.12)	<0.001
Cerebrovascular Disease	0.019 (-0.00 to 0.038)	0.06
Dementia	-0.075 (-0.12 to -0.03)	<0.001
Myocardial Infarction	0.016 (0.003 to 0.028)	0.013
Liver Disease	-0.073 (-0.086 to -0.059)	<0.001
Alcohol Abuse	0.026 (0.007 to 0.047)	0.007
Drug Abuse	-0.003 (-0.038 to 0.032)	0.876
Psychosis	0.011 (-0.010 to 0.032)	0.315
Depression	-0.018 (-0.033 to -0.003)	0016
Male (relative to female)	0.026 (0.025 to 0.027)	<0.001
Constant	0.026 (0.025 to 0.027)	<0.001
R^2	0.68	

[c]**eTable 2** provides some regression results from the regression equation used to estimate predicted case lengths. Details on this regression equation are provided in the **eMethods**. 95% confidence intervals shown in parentheses. Due to the extremely large number of variables, the coefficients for the surgery, facility, and age effects are not presented but are available on request.

eTable 3. Differences in Type of Institution Between Included and Excluded Cases[a]

© 2018 Sun EC et al. *JAMA Netw Open.*

	Included Cases (n=6,261,955)	Excluded Cases (n=13,955,260)	p
Institution Type (%)			
University Hospital	10.5 (10.5-10.5)	11.4 (11.1-11.5)	<0.001
Large Community Hospital	17.5 (17.5-17.5)	21.4 (21.4-21.4)	<0.001
Medium Community Hospital	43.2 (43.1-43.3)	40.4 (40.3-40.4)	<0.001
Small Community Hospital	3.6 (3.5-3.6)	4.1 (4.1-4.1)	<0.001
Specialty Hospital	3.7 (3.7-3.8)	2.0 (1.9-2.0)	<0.001
Attached Surgery Facility	5.0 (5.0-5.0)	5.5 (5.4-5.5)	<0.001
Freestanding Surgery Center	16.5 (16.5-16.6)	13.9 (13.8-13.9)	<0.001
Pain Clinic, Surgeon's Office, or Dental Office	0 (0-0)	1.4 (1.4-1.4)	<0.001

[a]**eTable 3** shows the percentage of cases performed at various surgical facility types (e.g. university hospital), stratified by cases in our final sample ("included cases") and cases that were excluded. 95% confidence intervals shown in parentheses. "p-value" shows the significance of differences between the two groups as assessed by a chi-square test. Note that the sum of included plus excluded cases (20,217,215) is less than our initial sample of 26,568,734 since some cases had missing information about facility type.

© 2018 Sun EC et al. *JAMA Netw Open.*

eTable 4. Distribution of Surgical CPTs Between Included and Excluded Cases[b]

	Included Cases (N=6,261,955))			Excluded Cases (n=13,292,917)		
Rank	CPT Code	Description	N (%)	CPT Code	Description	N (%)
1	66984	Cataract Surgery	276,408 (4.4%)	45378	Colonoscopy	606,862 (4.5%)
2	45378	Colonoscopy	234,874 (3.8%)	66984	Cataract Surgery	465,919 (3.5%)
3	43235	EGD	145,944 (2.3%)	43235	EGD	315,589 (2.4%)
4	43239	EGD	138,513 (2.2%)	43239	EGD	314,910 (2.4%)
5	90870	Electroconvulsive Therapy	132,867 (2.1%)	27447	Total Knee Arthroplasty	218,888 (1.6%)
6	47562	Cholecystectomy	130,210 (2.1%)	47562	Cholecystectomy	211,943 (1.6%)
7	59410	Vaginal Delivery	121,709 (1.9%)	45380	Colonoscopy	198,605 (1.5%)
8	27447	Total Knee Arthroplasty	117,589 (1.9%)	59409	Vaginal Delivery	190,360 (1.4%)
9	59409	Vaginal Delivery	112,440 (1.8%)	29881	Knee Arthroscopy	172,362 (1.3%)
10	29881	Knee Arthroscopy	105,513 (1.7%)	99231	Office Visit	141,405 (1.1%)
11	59514	Cesarean Section	86,404 (1.4%)	90870	Electroconvulsive Therapy	132,007 (1.0%)
12	69436	Myringotomy	85,802 (1.4%)	45385	Colonoscopy	129,573 (1.0%)
13	42820	Tonsillectomy	82,815 (1.3%)	27130	Total Hip Arthroplasty	123,154 (1.0%)
14	44970	Appendectomy	80,273 (1.3%)	59514	Cesarean Section	121,328 (0.9%)
15	45380	Colonoscopy	78,505 (1.3%)	64721	Neuroplasty	105,946 (0.8%)
16	27130	Total Hip Arthroplasty	72,485 (1.2%)	36620	Arterial Line Placement	104,245 (0.8%)
17	41899	Dental Surgery	63,661 (1.0%)	58558	Hysteroscopy	101,374 (0.8%)
18	49505	Hernia Repair	58,135 (0.9%)	44970	Appendectomy	98,221 (0.7%)
19	64721	Neuroplasty	57,242 (0.9%)	49505	Hernia Repair	96,122 (0.7%)
20	58588	Hysteroscopy	56,895 (0.9%)	29827	Knee Arthroscopy	92,094 (0.7%)

© 2018 Sun EC et al. *JAMA Netw Open.*

21	36620	Arterial Line Placement	53,589 (0.9%)	62311	Epidural Injection	89,196 (0.6%)
22	47563	Cholecystectomy	45,028 (0.7%)	69436	Myringotomy	80,237 (0.6%)
23	45385	Colonoscopy	42,667 (0.7%)	63030	Laminotomy	79956 (0.6%)
24	50590	Lithotripsy	42,085 (0.7%)	99100	Anesthesia Modifer Code	78791 (0.6%)
25	63030	Laminotomy	41,858 (0.7%)	42820	Tonsillectomy	75886 (0.6%)

[b]**eTable 4** shows the 25 most frequently surgical Current Procedural Terminology (CPT) codes reported for the anesthesia cases in our final sample ("included cases") and for excluded cases ("excluded cases") for which the surgical CPT code was reported. The table list the CPT code, a description of the procedure associated with the code, and the number (and percentage) of cases for which the code was reported. These 25 codes accounted for 33% of all cases for excluded cases and 39% of included cases. Data for other CPT codes is available from the authors on request.

© 2018 Sun EC et al. *JAMA Netw Open.*

© 2018 Sun EC et al. *JAMA Netw Open.*

eFigure. Distribution of Anesthesia Times

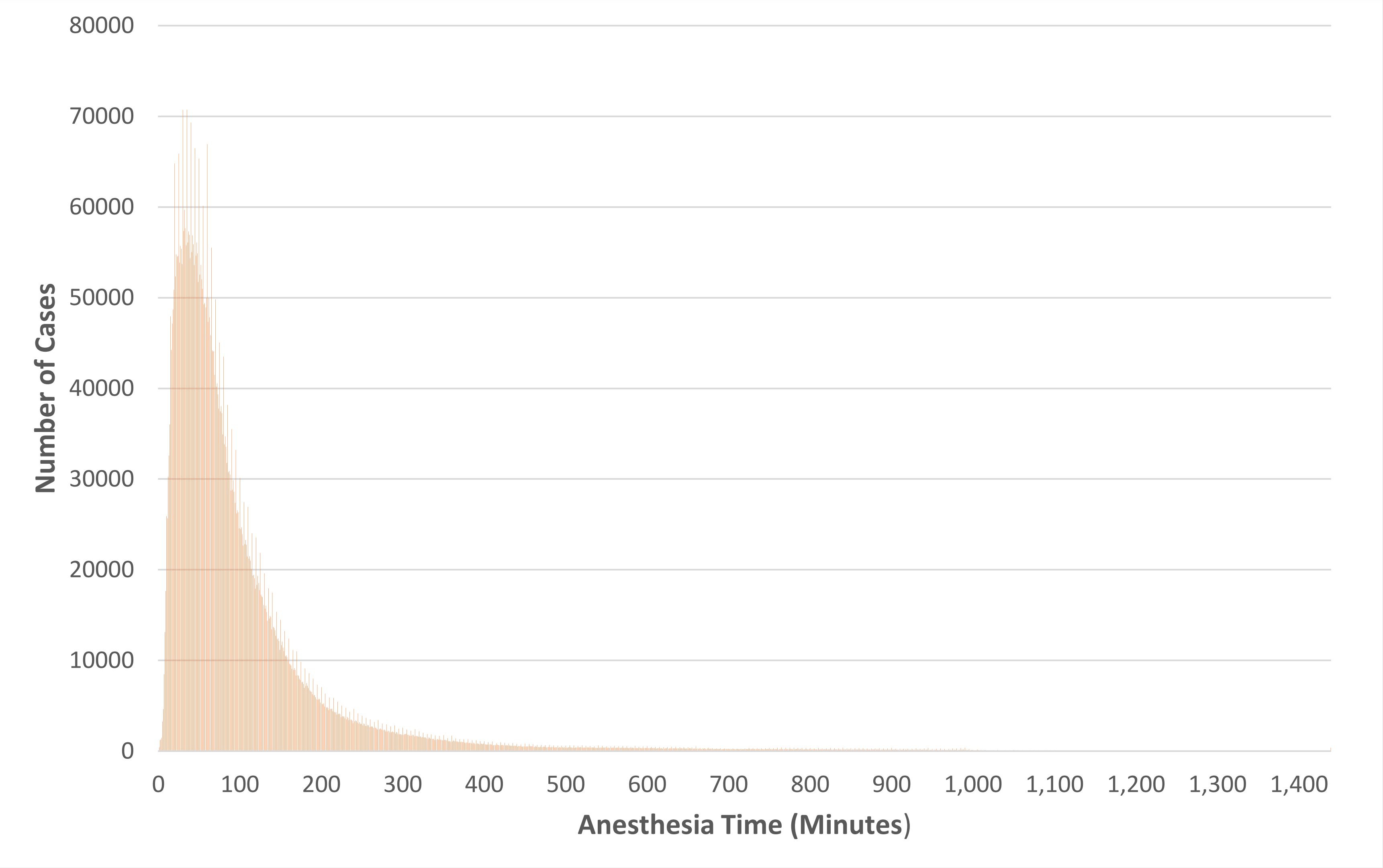

eFigure shows the distribution of anesthesia times for the 6,261,955 cases in our sample. The height for each column represents the number of cases with the reported anesthesia time (in minutes).

© 2018 Sun EC et al. *JAMA Netw Open.*

eMethods. Regression Model

This section outlines the regression model used to estimate the expected anesthesia times. Our regression model is given by the following equation:

$$ln(time_{ijkt}) = surgery_j + \delta_t + f_k + \Gamma X_i + \varepsilon_{ijkt} \quad (1)$$

The unit of observation for our analysis is the individual case (indexed by *i).* In the equation above, *j* is an index for each type of procedure (based on the surgical CPT code), *k* indexes the facility, and *t* indexes the year the case took place. $surgery_j$ is a fixed effect for the given surgical CPT, δ_t is a year effect, f_k is a facility fixed effect, and X_i is a vector of patient characteristics including age, gender, and the presence of the comorbidities outlined in Appendix Table 1. In effect, the regression equation above estimates the expected time for a given anesthesia case by estimating the average time, after adjusting for surgery type, the year the surgery was performed, the facility where the surgery was performed, and patient characteristics including age, gender, and comorbidities. In estimating the equation above, we take the natural log of anesthesia time since, as shown in figure 1, the natural distribution of anesthesia times broadly resembles a log normal distribution. Regression results from equation (1) are shown in **eTable 4.**

We can use the estimated regression coefficients from equation (1) to obtain the predicted time for a given case. Since our regression equation used the natural log of anesthesia time as the dependent variable, the predicted value from equation (1) is *approximately* the natural log of the predicted time. However, we use Duan's smearing estimator to convert the predicted values from the equation above into the actual predicted time (measured in minutes).

Having obtained the predicted times for a given case, we then calculate the difference between the expected and observed times for each case. We then estimate the following linear regression:

$$diff_i = \propto + \beta_1 Top5_i + \beta_2 Next5_i + \varepsilon_{ijkt} \quad (2)$$

where $diff_i$ is the difference between the observed and the expected time for case *i*, $Top5_i$ is an indicator variable that equals one if the anesthesia for case *i* was in the top 5th percentile (in terms of reporting anesthesia times ending in a multiple of 5), and $Next5_i$ is an indicator variable that equals one if the anesthesia practioner was in the top 6th to 10th percentile (in terms of reporting anesthesia times ending in a multiple of 5). β_1 and β_2 are our coefficients of interest, and represent the average amount by which observed anesthesia times for practitioners in these two groups exceed the expected times, compared to practitioners not in these two groups. For example, if β_1 equals 30, this would imply that observed times for anesthesia practitioners in the top 5th percentile exceed expected times by 30 minutes more compared to other anesthesia practitioners. In estimating equation (2) , we cluster our standard errors at the practitioner level.
